# PERSPECTIVE: Challenging preoperative α-blockade in phaeochromocytoma surgery: beyond tradition, towards ‘safer surgery’

**DOI:** 10.1530/EC-25-0139

**Published:** 2025-04-14

**Authors:** Isabelle Holscher, Koen M A Dreijerink, Markus W Hollmann, Tijs J van den Berg, Wouter D Lubbers, Anton F Engelsman, Els J M Nieveen van Dijkum

**Affiliations:** ^1^ Amsterdam UMC, Department of Surgery, Cancer Center Amsterdam, Amsterdam, The Netherlands; ^2^ Amsterdam UMC, Department of Endocrinology and Metabolism, Amsterdam, The Netherlands; ^3^ Amsterdam UMC, Department of Anesthesiology, Amsterdam, The Netherlands

**Keywords:** phaeochromocytoma, adrenalectomy, alpha-blockade, survey

## Abstract

The rarity of phaeochromocytomas has left a gap in evidence supporting guideline recommendations for preoperative α-blockade dose-escalation. Despite recent studies questioning its efficacy, randomized-controlled trials (RCTs) are warranted before considering omitting preoperative α-blockade dose-escalation. Through an online survey, opinions on the ideal study design for this future RCT were gathered from specialists involved in phaeochromocytoma management in the Netherlands. Responses from 23 physicians suggest a non-inferiority-designed RCT that only excludes patients with severe comorbidities and incorporates clinical outcome measures as most suitable design. The survey furthermore revealed diverse opinions regarding study design and perioperative threats, emphasizing the importance of an inclusive, multidisciplinary approach in future research.

The rarity of phaeochromocytomas has left a gap in evidence supporting existing guideline recommendations for preoperative α-blockade dose-escalation. While this pre-treatment aims to reduce cardiovascular complications, recent studies question its efficacy. The systematic review of Schimmack *et al.* (1) revealed no beneficial efficacy compared to no α-blockade and highlighted potential harm, including prolonged postoperative hypotension. The updated meta-analysis of Wang *et al.* (2) confirmed Schimmack’s conclusions, describing their findings as ‘a critical appraisal against the adherence to a dogmatic preparation paradigm, which is cumbersome, expensive and without evident benefits’.

Despite these outcomes, randomized-controlled trials (RCTs) are warranted before considering omitting preoperative α-blockade dose-escalation. However, besides practical challenges of conducting RCTs for rare diseases like phaeochromocytomas, there is also a lack of consensus on optimal study design for assessing perioperative outcomes, as evident in the current heterogeneity in research designs.

To determine this ideal RCT design, we conducted an online survey to gather opinions from specialists involved in phaeochromocytoma management in the Netherlands on preferred study designs, outcome measures and patient eligibility criteria.

The responses from 23 physicians (including eight surgeons, seven endocrinologists and seven anaesthesiologists) from eight centres (response rate of 71.9%) demonstrated the diverse opinions within the specialist community (Table 1). The most heterogeneous responses were regarding preferred primary endpoint; 43% (10/23) favoured surrogate endpoints, with the PRESCRIPT study’s haemodynamic instability (HDI) score (3) as most preferred endpoint. The remaining respondents argued that such metrics may lack clinical relevance and proposed the clinical outcomes of cardiovascular events and perioperative complications as more appropriate to assess the entire process – Dr Wang’s study (2) further debated the significance of intraoperative HDI as outcome due to a lack of data establishing a clear association with perioperative complications; intraoperative HDI is still common despite adequate α-blockade pre-treatment but rarely associated with significant cardiovascular events (2, 4, 5). This underscores the importance of utilizing clinical outcome measures into future RCTs and not focussing on HDI as primary endpoint.

**Table 1:** Survey responses.

Participant characteristics	
**Profession**	**Total**
Surgeon (surg)	**8 (35%)**
Internist (int)	**7 (30.5%)**
Anaesthesiologist (anes)	**7 (30.5%)**
Nurse-scientist	**1 (4%)**

Study design				
**Biggest perioperative threat**	**Int**	**Surg**	**Anes**	**Total**
Hypertension	7	6	4	**18 (78%)**
Hypotension		2	3	**5 (22%)**
**Preferred study design**	**Int**	**Surg**	**Anes**	**Total**
RCT with non-inferiority design	4	3	1	**9 (39%)**
RCT with non-inferiority design on primary outcome but superiority design on secondary outcomes	1	2	5	**8 (35%)**
Prospective observational with stepped wedge design		3	1	**4 (17%)**
RCT with superiority design	1			**1 (4%)**
RCT; design depending on endpoints	1			**1 (4%)**
**Preferred primary outcome measure**	**Int**	**Surg**	**Anes**	**Total**
Haemodynamic instability score (PRESCRIPT study)	4	3	2	**9 (39%)**
Cardiovascular events	3	1	2	**6 (23%)**
Time weighted average of hyper-/hypotension, brady-/tachycardia			1	**1 (4%)**
Hospital stay		2		**2 (9%)**
Complications overall		2	2	**5 (22%)**
**Exclusion of patients**	**Int**	**Surg**	**Anes**	**Total**
Yes	7	4	3	**15 (65%)**
No		4	4	**8 (35%)**

Furthermore, while hypertension consistently emerged as primary perioperative concern, significant variations in prioritization existed across different disciplines; 100% of endocrinologists prioritized hypertension, compared to 75% of surgeons and 57% of anaesthesiologists. This group perceived hypertension as a more hazardous and less manageable perioperative threat than hypotension. However, 22% (5/23) of the respondents who indicated hypotension as their primary concern stated that prolonged severe hypertension preceding diagnosis contributes to existing morbidity, making it less likely to manifest suddenly during surgery. In contrary, abrupt hypotension may be more detrimental, often requiring post-anaesthesia care unit intervention with haemodynamic support.

In terms of study design, 39% (9/23) favoured a non-inferiority RCT, while 35% (8/23) preferred a mixed-design RCT with non-inferiority for primary endpoints and superiority for secondary outcomes. Regarding patient eligibility, 65% (15/23) favoured excluding certain patients, with the most consensuses on excluding patients in phaeochromocytoma crisis (74%), patients with cardiac decompensation (78%) and those with a tumour size ≥6 cm (24%).

A limitation of our survey is the focus on Dutch specialists, which may affect generalizability. However, the equal inclusion of the different disciplines strengthens our findings’ robustness. The diverse perspectives regarding study design and perioperative threats, alongside the predominant monodisciplinary focus of current guidelines led by endocrinologists, emphasize the necessity of a multidisciplinary approach in future research, particularly when conducting RCTs.

In conclusion, recent studies highlight the evolving understanding of phaeochromocytoma management. More research, preferably RCTs, is needed to improve treatment strategies and patient outcomes. Our survey suggests a non-inferiority-designed RCT that only excludes patients with severe comorbidities and incorporates clinical outcome measures as most suitable design. We hope our findings contribute to this ongoing discussion and assist in shaping future RCT frameworks.

## Declaration of interest

The authors declare that there is no conflict of interest that could be perceived as prejudicing the impartiality of the work reported.

## Funding

This work did not receive any specific grant from any funding agency in the public, commercial or not-for-profit sector.

## Data availability

Data from this cohort study are available upon request from the corresponding author, Els J M Nieveen van Dijkum.

## Ethical approval

The paper is exempt from ethical committee approval because no patients were involved in the conduct of this study.
